# Health and social services integration at the local level: evidence and transition

**Published:** 2012-09-04

**Authors:** Peter Thistlethwaite

**Affiliations:** Director of Integrated Care R and D and Editor UK Journal of Integrated Care, UK

**Keywords:** integrated care, community health, social services, action research

## Abstract

Integration means change for professionals and for organisations: such change has frequently been resisted. Using a case study of the integration of community health and social services, the presentation will reflect on the way evidence and action research can be used to sustain change and help secure the benefits of integrated care for individuals, organisations and taxpayers.

Powerpoint presentation available at http://www.integratedcare.org at congresses – San Marino – programme.

